# Reducing PD-L1 expression with a self-assembled nanodrug: an alternative to PD-L1 antibody for enhanced chemo-immunotherapy

**DOI:** 10.7150/thno.45777

**Published:** 2021-01-01

**Authors:** Shuxian Cai, Ziyi Chen, Yingjie Wang, Min Wang, Junye Wu, Yuhong Tong, Lanlan Chen, Chunhua Lu, Huanghao Yang

**Affiliations:** MOE Key Laboratory for Analytical Science of Food Safety and Biology, Fujian Provincial Key Laboratory of Analysis and Detection Technology for Food Safety, State Key Laboratory of Photocatalysis on Energy and Environment, College of Chemistry, Fuzhou University, Fuzhou 350116, P. R. China.

**Keywords:** immune checkpoint therapy, repurposing old drug, immunoadjuvant metformin, reducing PD-L1 expression, self-assembled nanodrug

## Abstract

The binding between the immune checkpoints, programmed cell death ligand 1 (PD-L1) and programmed cell death 1 (PD-1), compromises T-cell-mediated immune surveillance. Immune checkpoint therapy using immune checkpoint inhibitors (ICIs) to block PD-L1 on cancer cell membrane or PD-1 on activated T cell membrane can restore antitumor function of T cell. However, the intracellular expression of PD-L1 and its active redistribution to cancer cell membrane may impair the therapeutic benefits of ICIs. To address this issue, herein we develop a nanodrug (MS NPs) capable of reducing PD-L1 expression and enhancing antitumor effects.

**Methods:** The nanodrug was self-assembled from immunoadjuvant metformin (Met, an old drug) and anticancer agent 7-ethyl-10-hydroxycamptothecin (SN38) via hydrogen bonds and electrostatic interactions. A series of experiments, including the characterization of MS NPs, the validation of MS NPs-mediated down-regulation of PD-L1 expression and *in vitro* therapeutic effect, the MS NPs-mediated *in vivo* chemo-immunotherapy and tumor metastasis inhibition were carried out.

**Results:** Different from ICIs that conformationally block PD-L1 on cancer cell membrane, MS NPs directly reduced the PD-L1 level via metformin to achieve immunotherapy. Therefore, MS NPs showed enhanced chemo-immunotherapy effect than its counterparts. MS NPs were also effective in inhibiting tumor metastasis by remodeling the extracellular matrix and restoring immune surveillance. Additionally, no obvious toxicity was observed in major organs from MS NPs-treated mice and a high survival rate of mice was obtained after MS NPs treatment.

**Conclusion:** We have designed nanodrug MS NPs by self-assembly of the immunoadjuvant Met and the anticancer agent SN38 for combined immunotherapy and chemotherapy. MS NPs might break the deadlock of antibody-based ICIs in immunotherapy, and repurposing old drug might provide a new perspective on the development of novel ICIs.

## Introduction

Programmed cell death ligand 1 (PD-L1) and its receptor, programmed death 1 (PD-1), are two typical immune checkpoints. The interaction between PD-L1 and PD-1 compromises T-cell-mediated immune surveillance, thus promoting cancer cells progression 1-3. Immune checkpoint therapy using immune checkpoint inhibitors (ICIs) to block PD-L1 on cancer cell membrane or PD-1 on activated T cell membrane can restore antitumor effect of T cell 4. ICIs has greatly improved the clinical outcomes of many advanced tumors 5,6. However, there are still some challenges of traditional ICIs for better treatment outcome 7,8. For example, ICIs have a risk of causing severe, even fatal immune-related adverse events (irAEs) that are harmful for other healthy organs 9-13. Besides, typical ICIs (antibody-based ICIs) need complicated production processes, high production costs and harsh storage conditions 14. Antibody-based ICIs achieve therapeutic effects by conformationally blocking PD-L1 on cancer cell surfac. This blocking effect is transient and could be weakened due to active redistribution of intracellular PD-L1 to cell membrane, resulting in impaired therapeutic effects 4,15,16. In light of these, it is urgent to develop ICIs with less side effects and enhanced antitumor performance by reducing endogeneous expression of PD-L1.

In recent decades, many old drugs with new uses have been reported for new drug discovery and development 17-19. For example, several chemotherapeutic agents that have been in use for a long time, such as doxorubicin, cyclophosphamide, and mitoxantrone, can evoke immunogenic cells death 20-22. Additionally, metformin (Met) is a prevalent drug for type 2 diabetes mellitus (T2DM) due to its effectiveness, safety, and cheap price 23. More and more studies have demonstrated that Met can curb tumor progression, improve the prognosis and survival rate of cancer patients 24-26. Furthermore, it has been reported that Met can induce abnormal PD-L1 glycosylation, leading to endoplasmic-reticulum-mediated degradation of PD-L1 and thus, facilitate antitumor immunity 27. These findings suggest that Met with new use may bring a glimmer of light to solve the dilemma of immune checkpoint therapy and pave the load for the discovery and development of new ICIs.

**Figure 1:**
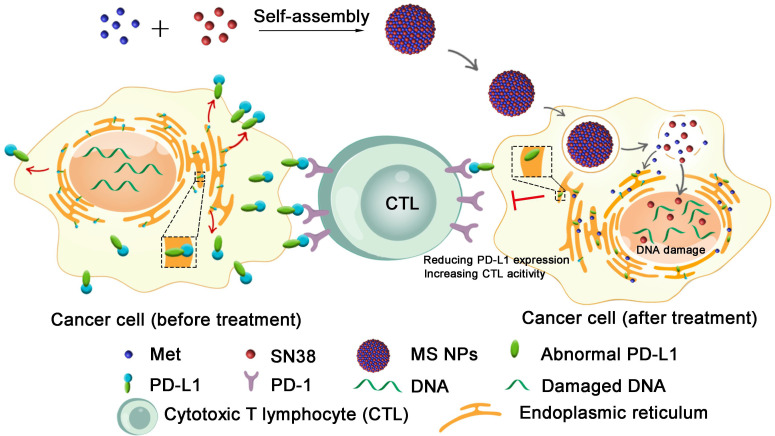
Illustration of reducing PD-L1 expression with self-assembled nanodrug for enhanced chemo-immunotherapy.

Herein, we described the development of a carrier-free nanodrug based on dual therapeutic agents self-assembly, which combines immunotherapy and chemotherapy for enhanced antitumor therapy (Figure 1). Met (served as immunoadjuvant) and anticancer drug 7-ethyl-10-hydroxycamptothecin (SN38, served as the DNA-topoisomerase I inhibitor) 28, were self-assembled into nanodrug (Met and SN38 self-assembled nanodrug denoted as MS NPs) via hydrogen bonds and electrostatic interactions. Different from traditional ICIs that only conformationally block cancer cells membranous PD-L1 in a transient manner, the proposed MS NPs degraded PD-L1 via Met for immunotherapy. Additionally, the nanodrug displayed improved metastasis inhibition as well as enhanced tumoricidal benefits, compared with the free therapeutic drugs. Moreover, MS NPs showed good biocompatibility, which may be due to carrier-free properties and the enhanced permeation and retention (EPR) effect of nanodrug 29,30. Collectively, nanodrug MS NPs might serve as a potential candidate for antibody-free ICIs and repurposing old drug offers a new idea for the development of novel ICIs.

## Results and Discussion

### Synthesis and characterization of self-assembled carrier-free nanodrug MS NPs

**Figure 2:**
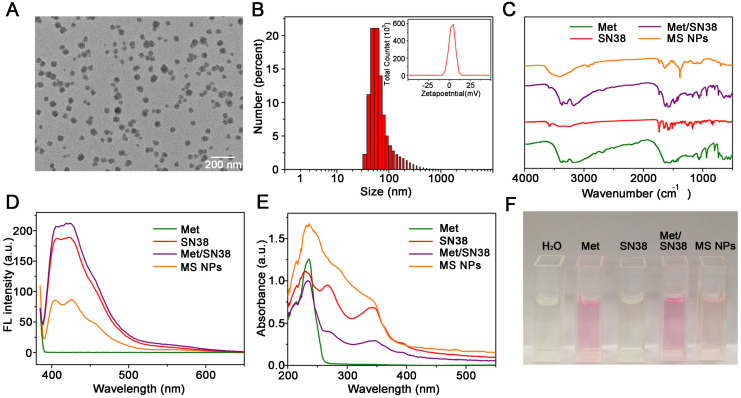
Characterizations of MS NPs. (A) TEM image of MS NPs. (B) Dynamic light scattering (DLS) measurement of MS NPs. Inset: The Zeta potential of MS NPs. (C) FTIR spectra, (D) Fluorescence spectra, (E) UV-vis absorption spectra, and (F) Sakaguchi reaction of MS NPs, Met, SN38 and Met/SN38.

The MS NPs were synthesized using a green and simple method in which Met and SN38 solutions were individually added dropwise to the double distilled water (DDW) with gentle agitation. The results of transmission electron microscopy image (TEM) showed that the synthesized MS NPs were round in shape with an average size of 50 nm (Figure 2A). In light of the cosolvent function of Met, MS NPs could be well-dispersed in DDW with a mean hydrodynamic diameter of approximately 90 nm (Figure 2B). The Zeta potential of MS NPs was evaluated to be 8.89 mV (Figure 2B Inset). And, MS NPs displayed good stability in 10% FBS for at least 3 days (Figure S1). The Fourier transform infrared (FTIR) spectrum of MS NPs showed a broad characteristic peak corresponding to the hydrogen bond about 3700-3000 cm^-1^ compared with that of Met 31,32, SN38 and the mixture of Met and SN38 (denoted as Met/SN38) (Figure 2C, Figure S2). As Met contains guanidyl, an amino-rich positively charged group, and SN38 owns negatively charged phenolic hydroxyl group, we assumed that Met and SN38 could self-assemble based on hydrogen bonds and electronic interactions. After exposure to a laser beam, MS NPs dissolved in DDW manifested a clear Tyndall effect, but this phenomenon vanished when MS NPs were dissolved in hydrogen bond-breaker dimethyl sulfoxide (Figure S3) 33. To validate the constituents of MS NPs, fluorescence spectral and UV-vis absorption spectral studies were carried out. The fluorescence spectrum of MS NPs displayed the characteristic fluorescence of SN38 (Figure 2D). Similarly, the UV-vis absorption spectrum of MS NPs exhibited the characteristic peaks of Met and SN38, which was indicative of the successful integrating of Met and SN38 (Figure 2E). In addition, Sakaguchi reaction and dark-box ultraviolet analysis were performed to further validate the components of MS NPs. Sakaguchi reaction is a typical color reaction for the detection of guanidyl 34. Therefore, only guanidyl-containing samples Met, Met/SN38 and MS NPs displayed positive results, characterized by a red color (Figure 2F). To visualize the fluorescence of samples, a dark-box UV analyzer was used. Both SN38 and Met/SN38 showed blue-purple fluorescence upon excitation at 365 nm by the dark-box ultraviolet analyzer (Figure S4). However, MS NPs displayed light yellow fluorescence, which may due to the presence of hydrogen bonds. As an important parameter, the drug loading ratio was determined. The Met and SN38 loading rates were calculated to be approximately 89.6% and 10.4%, respectively (Figure S5, S6). Taken together, these results indicate the successful self-assembly of nanodrug MS NPs consisting of Met and SN38.

### MS NPs-mediated down-regulation of PD-L1 expression

**Figure 3:**
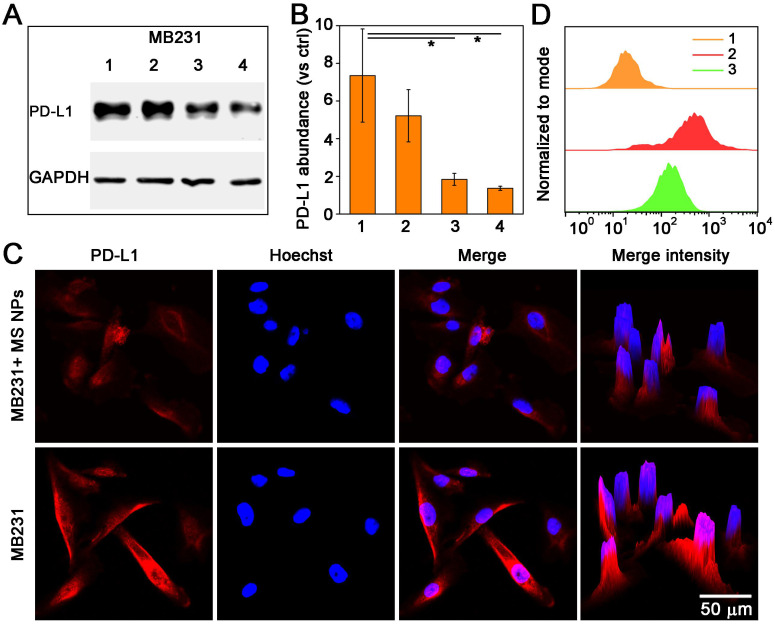
Decreased PD-L1 expression after treatment with MS NPs. (A) Western blot image of changes in PD-L1 level from MS NPs-treated MB231 cells. (Lane 1: MB231 cells, lanes 2-4: MB231 cells treated with different concentrations of MS NPs (20 µg/mL, 40 µg/mL, 80 µg/mL respectively.). (B) Densitometric analysis of PD-L1 bands from a. (C) CLSM images of PD-L1 expression in MB231 cells with (upper row) or without (bottom row) MS NPs treatment. (D) PD-L1 expression in MB231 cells with or without MS NPs treatment and measured by flowcytometry. Lines 1-3: MB231 cells, MB231 cells incubated with Alexa Fluor® 647 labelled anti-PD-L1, and MB231 cells treated with MS NPs and incubated with Alexa Fluor® 647 labelled PD-L1 antibody.

After confirming the formation of nanodrug MS NPs from Met and SN38, we then evaluated whether MS NPs could reduce the expression of PD-L1 in the human mammary breast cancer cell line, MDA-MB-231 (MB231), which overexpresses PD-L1. Firstly, western blot analysis was used to quantify the changes in the PD-L1 level. Western blot bands revealed that Met-containing samples can reduce PD-L1 expression in MB231 cells, while SN38-treated MB231 cells and untreated-MB231 cells expressed higher level of PD-L1 (Figure S7). The results of western blot and densitometric analysis results revealed the PD-L1 level was decreased dose-dependently in MB231 cells treated with MS NPs (Figure 3A-B). To visually observe the changes in PD-L1 expression, we used confocal laser scanning microscopy (CLSM) to image MB231 cells. After incubated with MS NPs and followed by Alexa Fluor® 647-conjugated PD-L1 antibody labeling, MB231 cells showed weak fluorescence, indicating that PD-L1 expression was attenuated upon MS NPs intervention (Figure 3C, upper row). By contrast, untreated MB231 cells presented brilliant fluorescence, which was indicative of high PD-L1 expression (Figure 3C, bottom row). Flow cytometry was used to further confirm the changes in PD-L1 level after MS NPs treatment (Figure 3D). The results showed that high PD-L1 expression in untreated MB231 cells but low expression in MS NP-treated cells. These findings strongly support the notion that the self-assembled nanodrug MS NPs can efficiently down-regulate PD-L1 expression. According to previous study, metformin can downregulate PD-L1 expression via activation of AMP-activated protein kinase (AMPK) and AMPK is locate in the ER region 27. To study the related downstream signaling pathway that MS NPs action, western blot assay was performed. As shown in Figure S8, MS NPs-treated MB231 cells showed up-regulated expression of phosphorylated AMPKα (Thr172) when compared with that of untreated-MB231 cells. To investigate the intracellular co-localization of MS NPs, FITC labelled MS NPs were firstly incubated with MB231 cells for 6 h, then cells were stained with ER-Tracker Red probe and Hoechst 33342. As shown in Figure S9, green fluorescence from FITC labelled MS NPs were in co-localization with red fluorescence from ER. And Pearson's correlation coefficient was calculated to be 0.81 indicating that a strong positive correlation in the intracellular distribution of MS NPs and ER-Tracker 35. Furthermore, line scanning profiles (Figure S9) exhibited that two kinds of fluorescence have the same trend, which further verifies the co-localization result. These results were in line with previous study that Met induces PD-L1 degradation via AMPK pathway locating in the ER 27.

### *In vitro* studies of MS NPs

Thereafter, the cellular uptake behavior of MS NPs was studied in MB231 cells by CLSM. The intracellular fluorescence intensity of MS NPs increased as incubation time extended (Figure S10), which can be due to the cellular uptake of more and more MS NPs by MB231 cells. Furthermore, a cell viability assay was performed to examine the *in vitro* therapeutic effect of MS NPs. In brief, MB231 cells were treated with Met, SN38, Met/SN38, or MS NPs for 24 h, and the viability of the cells was tested by the CCK-8 assay. As shown in Figure S11, the cells from the four groups exhibited concentration-dependent cytotoxicity. However, MS NPs-treated group showed better treatment outcome. When the incubation time was extended to 48 h, the therapeutic effects of MS NPs were concentration- and time-dependent (Figure S12). Taken together, these results confirm the enhanced anticancer activity of MS NPs.

### Blood retention and biodistribution studies of MS NPs *in vivo*

Given that nanoparticles with a suitable size usually have longer retention time in the bloodstream than free small-molecule drugs 36, we carried out the pharmacokinetic study by intravenous injection of the free Met and MS NPs to Balb/C mice. As shown in Figure S13, the concentration of MS NPs in the bloodstream was much higher than that of Met during 12 h. The blood clearance half time (t_1/2_) of MS NPs was 1.84-fold higher than that of free Met, confirming the good blood stability and pharmacokinetic profiles of MS NPs. Furthermore, the tissue distribution of MS NPs was examined in 4T1 tumor-bearing mice. When tumor volume reached approximately 100 mm^3^, the tumor-bearing mice were i.v. injection with free SN38 and MS NPs, respectively. SN38 treated group was used as control. The intratumoral SN38 concentration of MS NPs-treated mice reached 5.96 µg/g, which was 2-fold higher than that of free SN38-treated mice (Figure S14). Collectively, these results reflect that the nanodrug MS NPs facilitate the tumor accumulation of free SN38 via the nanoscale effect and longer bloodstream circulation time of MS NPs.

### *In vivo* combined chemotherapy and immunotherapy mediated by MS NPs

**Figure 4:**
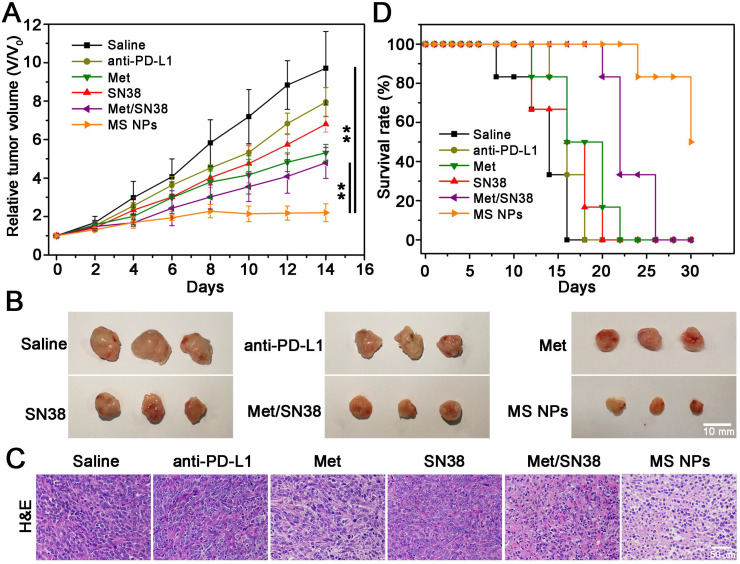
Combined chemo-immunotherapy of MS NPs. (A) Relative tumor volume (V/V_0_) curves of saline, anti-PD-L1, Met, SN38, Met/SN38, MS NPs treated groups. (B) Representative tumors *ex vivo* from mice after different treatments. (C) H&E staining of tumor tissues from mice with different treatments. (D) Survival rate of mice.

MS NPs-mediated degradation of PD-L1 and *in vitro* antitumor effect encouraged us to further explore their *in vivo* tumoricidal effect. When the tumor volume reached approximately 100 mm^3^, 4T1 tumor-bearing Balb/C mice were administered different drug formulations consisting of saline, ICI anti-PD-L1 antibody (anti-PD-L1), Met, SN38, Met/SN38 or MS NPs every 2 days for 12 days. Here, saline, anti-PD-L1, Met, SN38 and Met/SN38 injected mice were set as control groups. As showed in Figure 4A-B, saline group exhibited the fastest trend of tumor growth. Compared with mice from anti-PD-L1, Met, SN38 and Met/SN38 injection groups, the relative tumor volume of mice treated with MS NPs was obviously much smaller. After hematoxylin and eosin (H&E) staining, tumor tissue from MS NPs-treated revealed extensive cancer cells apoptosis characterized by deformed cells and condensed nuclei compared with control groups (Figure 4C). Hence, MS NPs provided benefits on inhibiting tumor growth. Survival rate analysis was also carried out to further validate the improved therapeutic effects of MS NPs. In agreement with relative tumor volume results, the survival rate of mice exposed to MS NPs was higher than that of other groups at 30 days (50% vs 0%) (Figure 4D). In addition, H&E staining of normal tissues revealed no apparent histopathological abnormality or lesion in the major organs from MS NPs group (Figure S15). Furthermore, no evident difference was observed in the body weight of mice treated with MS NPs compared with those treated with other formulations (Figure S16). These results indicate that MS NPs are effective in killing tumor cells with good safety.

**Figure 5:**
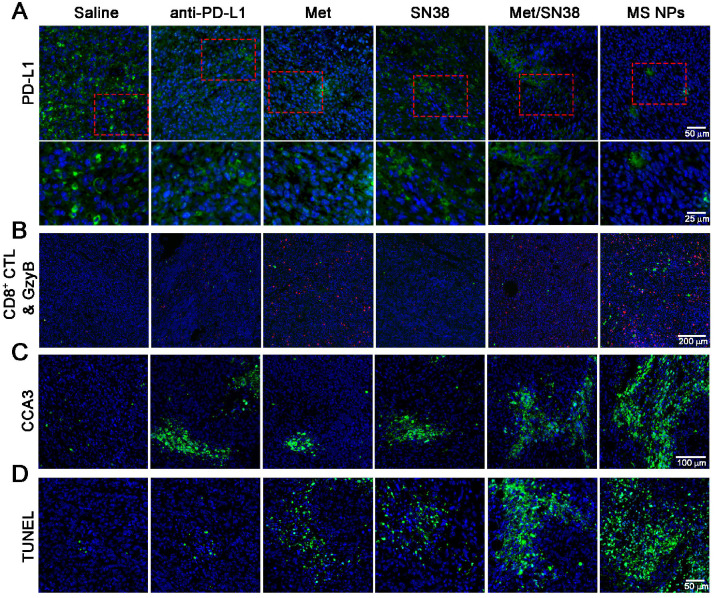
(A) Immunofluorescence staining of PD-L1 (green) (upper row) and the magnified images (bottom row), (B) CD8^+^ CTLs (red) and GzyB (green), (C) CCA3 (green), and (D) TUNEL (green) images. 4′,6-diamidino-2-phenylindole (DAPI) was used to stain cell nuclei (blue) in immunofluorescence assays.

To elucidate the antitumor effects integrating immunotherapy and chemotherapy, we firstly detected PD-L1 levels in tumor tissues from mice treated with the aforementioned entities. As shown in Figure 5A, PD-L1 expression was high in saline- and SN38-treated mice. However, the PD-L1 level was decreased in mice exposed to anti-PD-L1, Met, or Met/SN38 compared with those exposed to saline or SN38. The lowest PD-L1 level was observed in mice injected with MS NPs. To further verify that MS NPs-mediated down-regulated PD-L1 expression, qPCR was carried out. The qPCR data revealed that MS NPs can significantly reduce the expression of PD-L1 compared with other drugs (Figure S17). As the major force of antitumor immunity, CD8^+^ cytotoxic T lymphocyte (CD8^+^ CTL) can secrete granzyme B (GzyB) and interferon-γ (IFN-γ), to eradicate cancer cells. Therefore, the CD8^+^ CTL quantity and its activity, by detecting GzyB and IFN-γ release, were examined. Immunofluorescence and flow cytometry results indicated that MS NPs could effectively increase CD8^+^ CTL population (Figure 5B, Figure S18) and promote GzyB and IFN-γ releasing compared to control groups (Figure 5B, Figure S19). Because antitumor immunity is of concomitance with apoptosis, the cleaved caspase-3 (CCA3) level was measured. The results indicated extensive apoptosis in tumor tissue of MS NPs-treated mice. However, weak apoptotic signals were observed in tumor tissues of the controls (Figure 5C). In addition, chemotherapeutic drug SN38 is the inhibitor of DNA-topoisomerase I; thus, it can induce DNA strand damage. Thereafter, a TUNEL assay was performed to assess the degree of DNA strand damage. Although Met-, SN38- or Met/SN38-treated groups exhibited a fraction of cells underwent DNA damage, yet evident clustered DNA damage signals were extensively observed in MS NPs-treated group (Figure 5D). Together, these results support the fact that nanodrug MS NPs integrate immunotherapy and chemotherapy for tumoricidal purpose. It should be noted that PD-1 and PD-L1 blockade elicited CD8^+^ CTL and NK cell response 37,38. Our results showed that MS NPs can active PD-1 expressing CD8^+^ CTL by down-regulating PD-L1, restoring its tumor killing effect. Therefore, we speculate that NK cell expressing PD-1 might have a similar effect on MS NPs stimulation.

### *In vivo* tumor metastasis inhibition mediated by MS NPs

**Figure 6:**
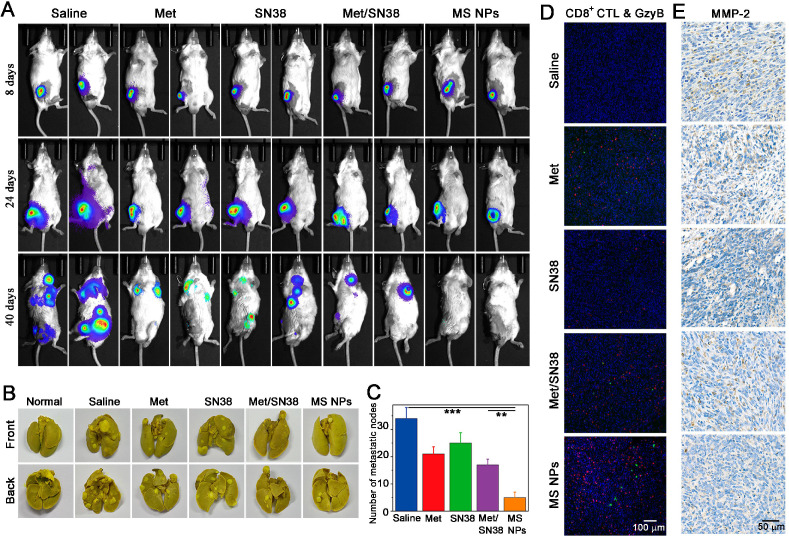
(A) Tumor metastasis inhibition with MS NPs. (A) *In vivo* bioluminescence images of spontaneous tumor metastasis of Balb/C mice bearing Luc-4T1 tumor with different treatments. (B) Representative images of metastatic nodules from lungs. (C) The number of metastatic nodules of *ex vivo* lungs (n=3). (D) Immunofluorescence images of CD8^+^ CTL and GzyB from tumor mass. (E) Immunohistochemistry images of MMP-2 from tumor mass.

A key step in tumor metastasis is the remodeling of extracellular matrix via the overexpression of matrix metalloproteinases, such as matrix metalloproteinase-2 (MMP-2) and matrix metalloproteinase-9 (MMP-9) 39-41. The elevation of MMPs can degrade extracellular matrix, thereby, promoting the metastasis of malignant cells. According to clinical report, metastasis accounts for approximately 90% of cancer-associated mortality 42. Thus, inhibiting tumor metastasis, as well as destroying the primary tumor is critical for effective cancer therapy. It has been reported that cancer immunotherapy can awaken the host's own immune system battle against metastasis. Coincidentally, Met could not only reduce the expression of PD-L1 but also decrease the expression of MMP-2 43. As a result, we explored whether MS NPs could inhibit tumor metastasis. A highly metastatic breast tumor model was established by planting highly metastatic murine 4T1 breast adenocarcinoma cells expressing firefly luciferase (Luc-4T1) into the subcutaneous of Balb/C mice. When tumor volume reached approximately 50 mm^3^, mice were exposed to different formulations including saline, Met, SN38, Met/SN38 or MS NPs. Saline, Met, SN38 and Met/SN38 injected mice were set as control groups. At different timepoints, mice were intraperitoneally injected with D-luciferin sodium salt (the substrate of luciferase), and *in vivo* bioluminescence images of spontaneous tumor metastasis were captured. As shown in Figure 6A, mice of the saline group exhibited evident metastasis signals on day 24, and obvious, uncontrolled multiple metastatic foci on day 40. Mice treated with Met, SN38 or Met/SN38 displayed clearly visible metastatic foci on day 40. By contrast, no obvious metastatic tumor signal was observed in MS NPs group on day 40. Compared with the controls, MS NPs group alleviated tumor metastasis the most effective. In line with these findings, control groups showed higher numbers of metastatic nodules in *ex vivo* lungs than that of MS NPs-treated group (Figure 6B-C). The mechanisms underlying these observed phenomena were investigated by detecting CD8^+^ CTL population as well as the levels of GzyB and MMP-2 in tumors from different groups. The results showed that MS NPs-treated group elevated the CD8^+^ CTL population and their activity (as indicated by GzyB level) to the most compared with the controls (Figure 6D). Moreover, the most significant decrease in MMP-2 expression was noted in the MS NPs-treated group (Figure 6E). These data suggest that MS NPs can effectively inhibit tumor metastasis, possibly by remodeling the extracellular matrix and evoking the immune system.

### *In vivo* toxicology evaluation of MS NPs

The potential long-term *in vivo* toxicity of MS NPs was evaluated by blood routine assay and blood chemistry for mice after intravenous injection with MS NPs (24 mg/kg). Saline treated mice were set as control group. All mice were killed at 7 and 14 days after injection for blood collection. Blood routine assay showed that all data for MS NPs treated mice were all in the normal ranges compared with those of saline treated mice (Figure S22). The levels of vital liver function markers, including alanine aminotransferase (ALT), aspartate aminotransferase (AST) and alkaline phosphatase (ALP), in the blood at different time points after MS NPs treatment appear to be normal (Figure S23A-C) 44,45. And the urea levels, the indicator of kidney functions, in the blood of MS NPs treated mice were also within reference range (Figure S23D) 44-46. Taken together, no appreciable systemic *in vivo* toxicity induced by MS NPs was detected at treatment dose within 14 days.

## Conclusions

In summary, we have designed nanodrug MS NPs by self-assembly of the immunoadjuvant Met and the anticancer agent SN38 for combined immunotherapy and chemotherapy. The results showed that MS NPs could achieve better tumoricidal effects than its individual counterparts. MS NPs were also effective in inhibiting tumor metastasis by remodeling the extracellular matrix and restoring immune surveillance. More importantly, the introduction of Met endowed MS NPs with the ability to decrease PD-L1 expression in cancer cells, thereby effectively awakening the host's immune system compared with the antibody drug anti-PD-L1 that function by conformational blockade on membranous PD-L1. In light of these findings, MS NPs might overcome the disadvantages of antibody-based ICIs and repurposing old drug might provide new insights for the development of novel ICIs.

## Materials and Methods

### Reagents and material

Metformin hydrochloride and SN38 were purchased from Sigma Aldrich (USA). BCA protein quantitation kit and normal goat serum were obtained from Solarbio Life Sciences (Beijing, China). ELISA kits (IFN-γ) were purchased from Elabscience Biotechnology Co., Ltd. Anti-PD-L1 was obtained from BioXCell. ER-Tracker Red probe was purchased from Shanghai Maokang Biotechnology Co., Ltd. Other reagents were obtained from standard suppliers and used as received.

### Synthesis of MS NPs

Briefly, Met (0.21 mg/mL) and SN-38 (0.5 mg/mL) were dissolved in ethyl alcohol (ETOH) respectively to prepare stock solutions. Then 1 mL of each solution was injected into 100 mL DDW (with gentle agitation) at flow rate of 20 µL/min through two separated channels of syringe pump (LSP02-1B, Longer Pump Co., Ltd, Baoding, Hebei, China.). After the injection, reactions lasted for another 15 min. Afterward, the solution was centrifugated and obtained precipitate was dialyzed (MWCO 8000-14000) in DDW to completely remove ETOH and free drugs. The obtained MS NPs were stored in the dark at 4 °C before use.

### Characterization of MS NPs

The morphology of MS NPs was characterized by TEM (Hitachi HT7700) with an acceleration voltage of 200 kV. The size distribution of MS NPs was measured by Zetasizer (Malvern Instruments). FTIR, fluorescence spectrophotometer and UV-visible spectrophotometer were used to confirm the successful self-assembly of Met and SN38. Sakaguchi reaction was conducted as described below. Different samples (Met, SN38, Met/SN38 and MS NPs) were mixed with the mixture of DDW and 1% α-naphthol ethanol solution, respectively, then hypobromite solutions were added with shaking. After 1 min, 10% NaOH was inhaled to the above solution, and color changes were observed. A dark-box UV analyzer was used to visualize the fluorescence of SN38-containing samples. The drug loading ratio of Met and SN38 were measured using HPLC and HPLC-MS, respectively. Standard curves of Met and SN38 have been showed in Figure S5-S6.

### Cells culture and animal feeding

Cells lines MB231 and 4T1 were purchased from the American Type Culture Collection (Manassas, VA). Luc-4T1 was purchased from Cobioer Biosciences Co., Ltd (Nanjing, China). MB231 and 4T1 were cultured in RPMI 1640 medium containing 10% FBS, 100 µg/mL streptomycin and 100 U/mL penicillin at 37 °C in a humidified atmosphere of 5% CO_2_. Luc-4T1 was cultured in RPMI-1640 medium supplemented with 10% FBS, 100 µg/mL streptomycin, 100 U/mL penicillin and puromycin (1 µg/mL) at 37 °C in a humidified atmosphere of 5% CO_2_. Female Balb/C mice (age, 6-8 weeks; weight, 18-20 g) were provide by Charles River (Charles Rive Laboratory Animal, Beijing, China). Protocols of animal experiments were performed according to the Guidelines for Care and Use of Laboratory Animals (Ministry of Science and Technology of China, 2006) and were approved by the Animal Ethics Committee of Fujian Medical University.

### Western blot and flow cytometric analysis

After treated with MS NPs for 48 h, MB231 cells were washed with cold PBS and lysed in lysis buffer (1× phosphatase inhibitor cocktail, and RIPA) on ice bath. Then cells lysates were collected with a cells-scraper and centrifuged (14,000 rpm at 4 °C for 15 min), and the supernatant was collected for protein quantitation. Proteins were separated by 10% SDS-PAGE and subsequently transferred to a polyvinylidene difluoride (PVDF) membrane (Bio-Rad, USA). The membrane was blocked with 5% BSA in TBST. For identifying PD-L1, the membrane was incubated with primary antibody for PD-L1 (Cells Signaling Technology) or GAPDH (Cells Signaling Technology) overnight at 4 °C. After being washed with TBST, the membrane was incubated with HRP-conjugated anti-rabbit secondary antibody, and reacted with chemiluminescent substrate. Then reactive bands were obtained using a ChemiDocTM Touch gel imaging system (Bio-Rad, USA). As for densitometric analysis, all PD-L1 bands were normalized to the internal reference GAPDH, respectively. Image Lab software (Bio-Rad, USA) was used for densitometric analysis.

Post-treatment, MB231 cells were collected and blocked with 10% normal goat serum at 4 °C for 1h. Then MB231 cells suspension was labelled with anti-PD-L1 antibody (Alexa Flor® 647, Abcam) at 4 °C for 30 min. After thoroughly washed with washing buffer (cold PBS containing10% normal goat serum), MB231 cells were measured with a Canto II flow cytometry (BD Bioscience, CA) equipped with a 633 nm laser. FlowJo software was used to analyze the data.

### Confocal laser scanning microscopy imaging

MB231 cells seeded on confocal dishes were exposed to MS NPs for 48 h. After fixation (4% formaldehyde, 10 min), permeabilization (0.1% TritonX-100, 5 min) and blocking (10% normal goat serum in 0.1% PBS-Tween, 1 h), the cells were stained with anti-PD-L1 antibody (Alexa Flor® 647, Abcam) overnight at 4 °C. Cells were incubated with Hoechst 33342 for 10 min to indicate cell nucleus, followed by washing with PBS and CLSM image.

### Cellular uptake of MS NPs

The cellular uptake of MS NPs was studied in MB231 cells using CLSM. MB231 cells seeded on confocal dishes were incubated with MS NPs for 2 h, 4 h, 6 h. Subsequently, cells were washed with PBS three times and observed with CLSM.

### *In vitro* treatment evaluation of MS NPs

CCK-8 assay was used to study the cytotoxicity of MS NPs against MB231 cells. MB231 cells were seeded on 96-well dishes at a density of 1×10^4^ cells per well. After settle down overnight, the cells were cultured with various drug formulations including Met, SN38, Met/SN38 or MS NPs in new media. Cells were incubated for 24 h. As for concentration-dependent and time-dependent cytotoxicity of MS NPs, the incubation time was set as 24 h and 48 h. The viability of cells was measured via CCK-8 assay kit.

### *In vivo* treatment evaluation of MS NPs

4T1 cells (2×10^6^) were inoculated subcutaneously into right legs of female Balb/C mice. When tumor volume reached ~100 mm^3^, female Balb/C mice were randomly divided into 6 groups (n=6). The first group of mice was injected with saline, as saline group; the second group was injected with anti-PD-L1 (2.0 mg/kg), as anti-PD-L1 group; the third group was injected with Met (11 mg/kg), as Met group; the forth group was injected with SN38 (1.1 mg/kg), as SN38 group; the fifth group was injected with Met/SN38, as Met/SN38 group (11 mg/kg Met and 1.1 mg/kg SN38); the sixth group was injected with MS NPs (12 mg/kg), as MS NPs group. Anti-PD-L1 was administered through intraperitoneal injection, other formulations were intravenously injected via tail vein once every other day for 12 days. The first administration was recorded as 0 day. The tumor volume was measured with a caliper every 2 days for 16 days, and relative tumor volume (V/V_0_) was calculated as the result. Body weight was measured every 2 days for 16 days. After the last measurement, mice were euthanized, tumor and major organs were collected for H&E staining.

### Survival rate analysis

36 female Balb/C mice bearing 4T1 tumors (about 100 mm^3^) were randomly divided into 6 groups (n=6) and received different treatments including saline, anti-PD-L1, Met, SN38, Met/SN38 or MS NPs. A total of 6 injections were conducted. The tumor volume was measured every 2 days. The first administration was recorded as 0 day. A mouse was defined as dead when its tumor volume reached 1000 mm^3^. And survival rate is equal to the number of surviving ones divided by the total number of mice for each group.

### Immunofluorescence

Tumor mass from mice was collected and frozen for cryostat section. After permeabilization and blocking, cryostat sections were incubated with the primary antibodies for PD-L1 (Abcam), CD8^+^ CTL (Cell Signaling Technology), GzyB (Cell Signaling Technology), active caspase 3 (Cell Signaling Technology) and TUNEL (Roche) overnight at 4 °C. After being washed with PBS for three times, these cryostat sections were incubated with corresponding secondary antibodies at room temperature for 1 hour. For nuclear staining, sections were incubated with DAPI. Then CLSM was used for photo imaging.

### T cell infiltration analysis with flow cytometry

T cell infiltration was analyzed with flow cytometry. Tumor tissues from mice with different treatments were collected and digested into single cell suspension. Then cell suspension was incubated with APC anti-mouse CD3^+^ antibody (Biolegend) and PE anti-mouse CD8^+^ antibody (Biolegend) and analyzed with flow cytometry.

### Cytokine detection

Tumor tissues were collected from mice after different treatments and homogenized. After centrifugation, the supernatant was collected and the cytokine of IFN-γ was detected by enzyme-linked immunosorbent assay (ELISA) according to manufacturer's instructions.

### Pharmacokinetics profile and biodistribution studies of MS NPs *in vivo*

To study the pharmacokinetics of MS NPs, Balb/C mice (n=3) were i.v. injection with Met or MS NPs at an identical Met dose of 11 mg/kg. Blood samples were taken from the eye socket at 5 min, 15 min, 30 min, 1 h, 2 h, 4 h, 8 h and 12 h after injection. After centrifugation at 3000 rpm for 15 min, the plasma was obtained. Then acetonitrile, protein precipitator, was added into plasma (2:1 v/v), and the solutions separated by centrifugation were collected. The blood concentration of Met and MS NPs were obtained using Xevo TQ-S. To investigate the tissue distribution of free SN38 and MS NPs, 4T1 tumor-bearing mice (n=3) were i.v. injection with free SN38 or MS NPs at an identical SN38 dose of 1.1 mg/kg, respectively. Mice were sacrificed to collect major tissues (heart, lung, spleen, liver, kidney and tumor) at 24 h after injection. After being homogenized, protein precipitated and filtered, these tissues were quantitatively detected by Xevo TQ-S to obtain the amount of SN38 and MS NPs.

### Metastasis inhibition evaluation

Luc-4T1cells (5×10^5^) were inoculated subcutaneously into right legs of female Balb/C mice. When tumor volume reached ~50 mm^3^, mice were randomly divided into 5 groups (n=6): (1) saline group; (2) Met group; (3) SN38 group; (4) Met/SN38 group; (5) MS NPs group. Drugs were intravenously injected via tail vein once every other day for 12 days. To relief the burden of mice, we removed the primary tumor of mice on the 30^th^ day. The Ami X optical imaging system (Spectral Instruments Imaging Co., USA) was used to image spontaneous metastases of cancer cells on days 8, 24, 40. Before *in vivo* bioluminescence images, D-luciferin sodium salt (150 mg/kg) was given via intraperitoneal injection. After the last imaging, the lungs and tumor of mice were removed. To examine lung metastasis, we took the picture of *ex vivo* of lungs and counted the number of lung nodules numbers.

### Immunohistochemical staining

In brief, tumor specimens were blocked with 3% BSA at room temperature for 30 min, then incubated with primary antibody against metalloproteinase-2 (MMP-2). After washing, samples were incubated with secondary antibody and visualized with DBA solution. The photographs of samples were taken using inverted fluorescence microscope (NIKON ECLIPSE Ti, Japan).

### Statistical Analysis

Statistical analysis of data were carried out using the Student's *t*-test. All results were showed as mean ± standard error. * *P* < 0.05, ** *P* < 0.01, *** *P* < 0.001.

## Supplementary Material

Supplementary figures.

## Abbreviations

PD-L1: programmed cell death ligand 1
PD-1: programmed cell death 1
ICIs: immune checkpoint inhibitors
irAEs: immune-related adverse events
MS NPs: Met and SN38 self-assembled nanodrug
Met: Metformin
T2DM: type 2 diabetes mellitus
EPR: the enhanced permeation and retention
DDW: double distilled water
TEM: transmission electron microscope
FTIR: Fourier transform infrared
DLS: Dynamic light scattering
MB231: MDA-MB-231
CLSM: confocal laser scanning microscopy
CCK-8: cell counting kit-8
anti-PD-L1: anti-PD-L1 antibody
H&E: hematoxylin and eosin
CD8^+^ CTL: CD8^+^ cytotoxic T lymphocyte
GzyB: granzyme B
CCA3: cleaved caspase-3
DAPI: 4′,6-diamidino-2-phenylindole
MMP-2: metalloproteinase-2
MMP-9: matrix metalloproteinase-9
Luc-4T1: 4T1 breast adenocarcinoma cells expressing firefly luciferase
