# Supplementary material for: Reducing PD-L1 expression with a self-assembled nanodrug: an alternative to PD-L1 antibody for enhanced chemo-immunotherapy
Source: Theranostics. 2021 Jan 1;11(4):1970–81. doi: 10.7150/thno.45777 (PMC7778587; doi:10.7150/thno.45777)
Supplement: Supplementary file 1 — Supplementary figures. [file thnov11p1970s1.pdf]

Supplementary Information

Reducing PD-L1 expression with a self-assembled nanodrug: an alternative to PD-L1 antibody for enhanced chemo-immunotherapy

Shuxian Cai, Ziyi Chen, Yingjie Wang, Min Wang, Junye Wu, Yuhong Tong, Lanlan Chen✉, Chunhua Lu✉ and Huanghao Yang✉

MOE Key Laboratory for Analytical Science of Food Safety and Biology, Fujian Provincial Key Laboratory of Analysis and Detection Technology for Food Safety, State Key Laboratory of Photocatalysis on Energy and Environment, College of Chemistry, Fuzhou University, Fuzhou 350116, P. R. China.

✉ Corresponding Author: llchen@fzu.edu.cn; chunhualu@fzu.edu.cn; hhyang@fzu.edu.cn.

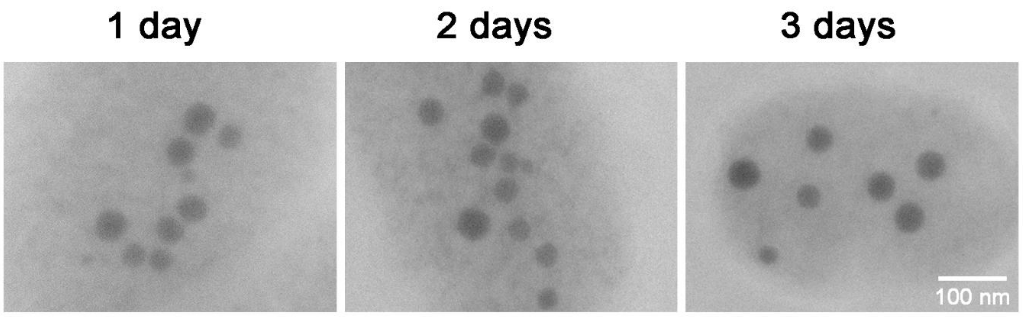

Figure S1. TEM images of MS NPs stability. To evaluate the stability, MS NPs was dissolved in 10% FBS solution and TEM was used to photograph its morphology at preset timepoints.

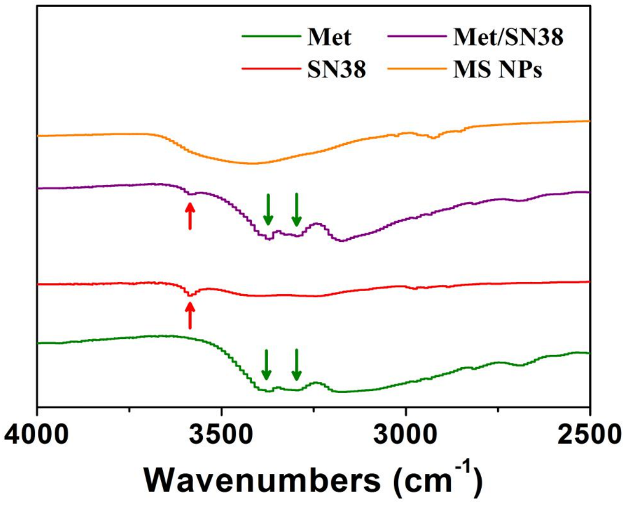

Figure S2. Magnified FTIR spectra of Met, SN38, Met/SN38 and MS NPs. The red arrows indicate the hydroxyl group from SN38, and the green ones indicate the amino group from metformin.

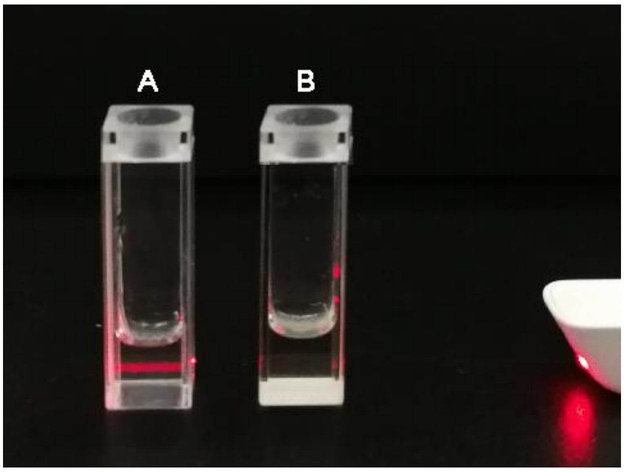

Figure S3. Tyndall effect of (A) MS NPs in double distilled water, (B) MS NPs in DMSO.

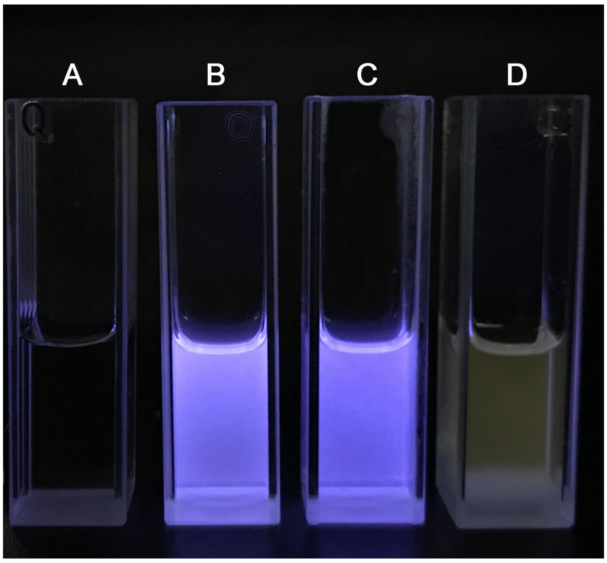

Figure S4. Photographs of (B) Met, (C) SN38, (D) Met/SN38, (E) MS NPs when excited at 365 nm in dark-box ultraviolet analyzer.

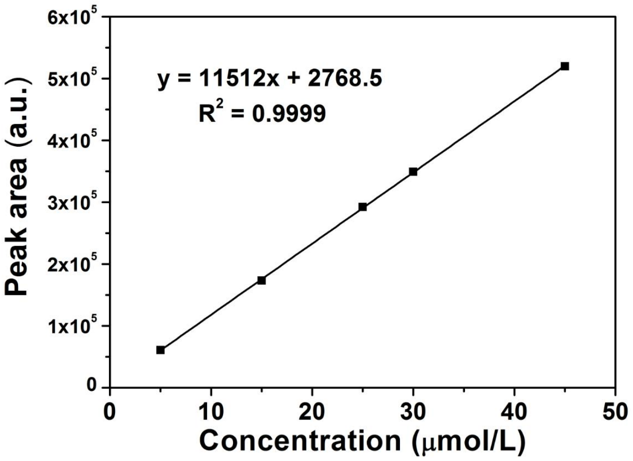

Figure S5. Standard curve of metformin.

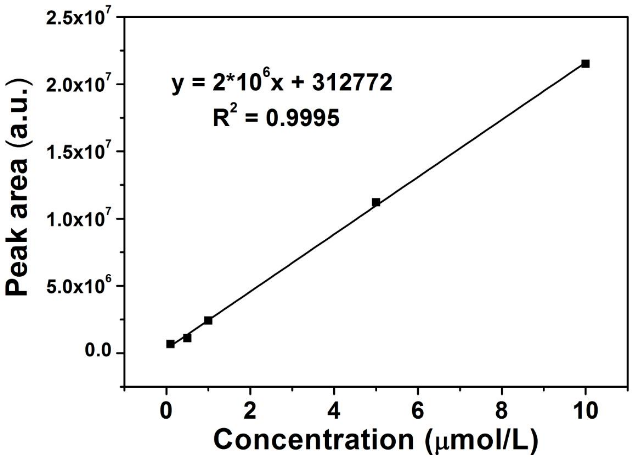

Figure S6. Standard curve of SN38.

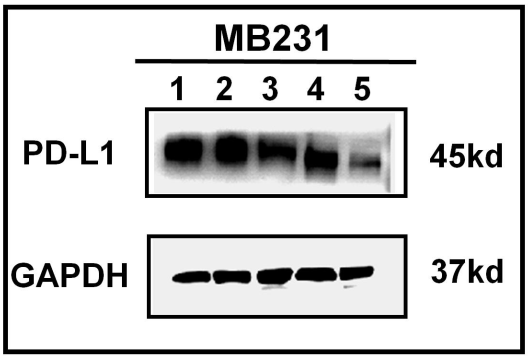

Figure S7. Western blot assay of PD-L1 expression of MB231 cells after treatment with (lane 2) SN38, (lane 3) Met, (lane 4) Met/SN38 and (lane 5) MS NPs, respectively. Lane 1 corresponds to untreated MB231 cells.

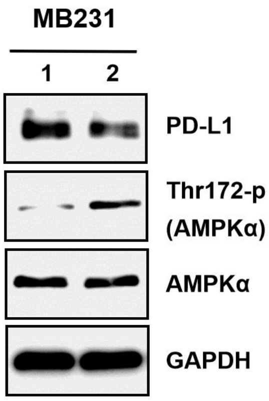

Figure S8. Western blot assay of PD-L1 and phosphorylated AMPKα (Thr172p) expressions of MB231 cells after treatment with MS NPs (lane 2). Lane 1 corresponds to untreated MB231 cells.

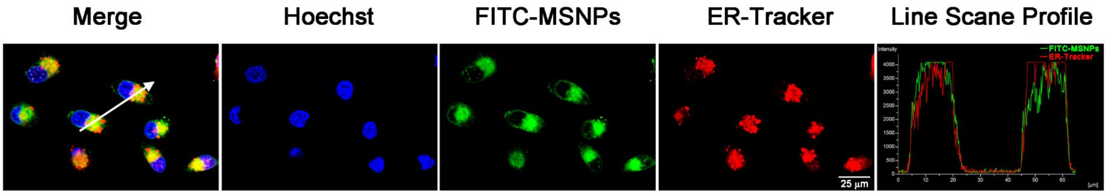

Figure S9. Intracellular co-localization of MS NPs. CLSM images of live MB231 cells treated with FITC labelled MS NPs (pseudocolored in green). Cell nucleus and endoplasmic reticulum (ER) were stained with Hoechst 33342 (pseudocolored in blue) and ER-Tracker Red probe (pseudocolored in red), respectively. Line scanning profiles corresponded to the fluorescence of FITC labelled MS NPs and ER. Scale bars: 25 μm.

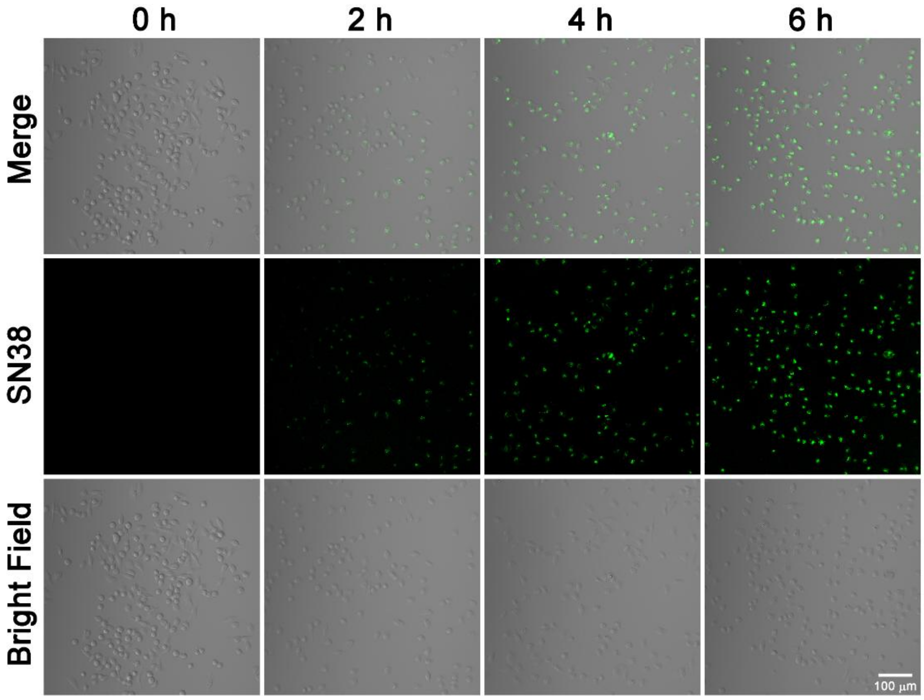

Figure S10. Time-dependent cellular uptake of MS NPs in MB231 cells. Scale bars: 100 μm.

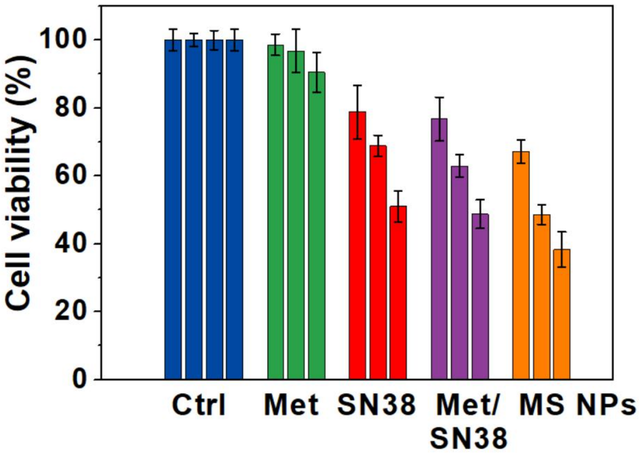

Figure S11. Cytotoxicity of Met, SN38, Met/SN38 and MS NPs treated MB231cells for 24h. MB231 cells without treatment was set as controls.

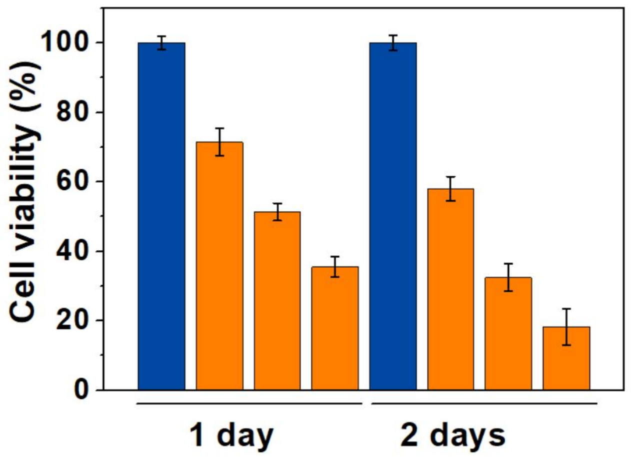

Figure S12. Cytotoxicity of MS NPs (orange) at different concentrations and incubation times. MB231 cells without treatment was set as controls (blue).

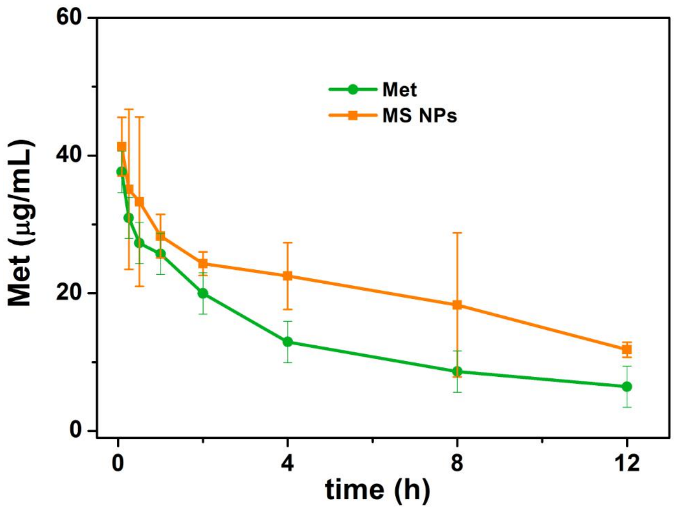

Figure S13. Typical plasma concentration-time profiles of free Met and MS NPs after intravenous injection into mice.

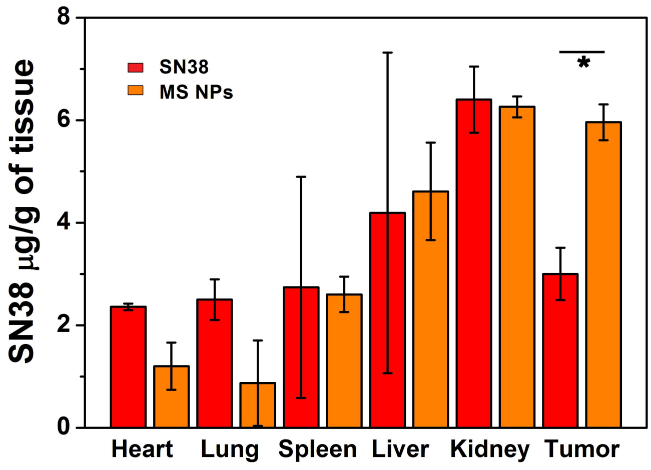

Figure S14. Tissue distribution of SN38 and MS NPs after intravenous injection of freeSN38 and MS NPs in mice.

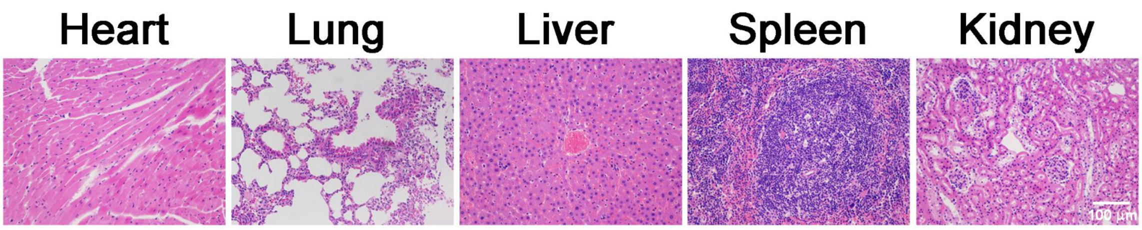

Figure S15. H&E staining images of major organs collected from MS NPs treated mice.

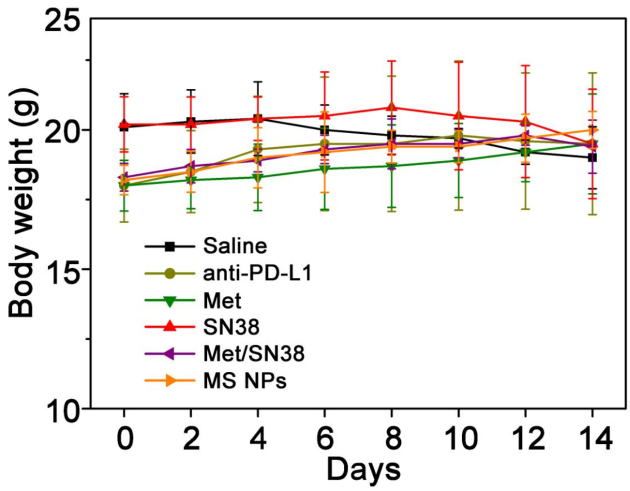

Figure S16. Body weights of saline, anti-PD-L1, Met, SN38, Met/SN38 and MS NPs treated mice.

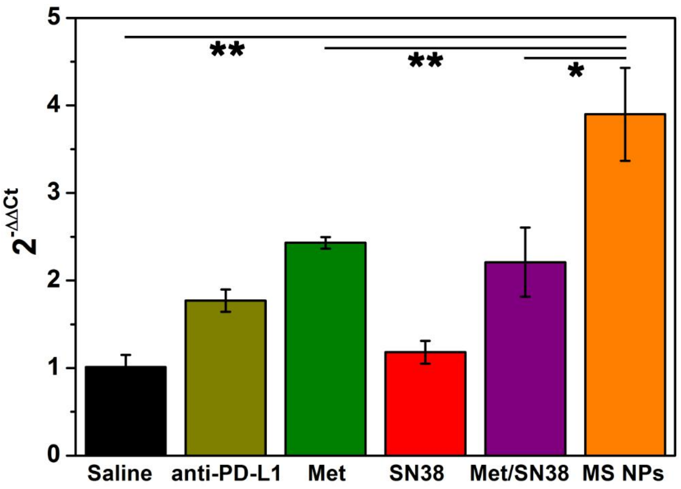

Figure S17. The qPCR results of saline, anti-PD-L1, Met, SN38, Met/SN38 and MS NPs treated mice.

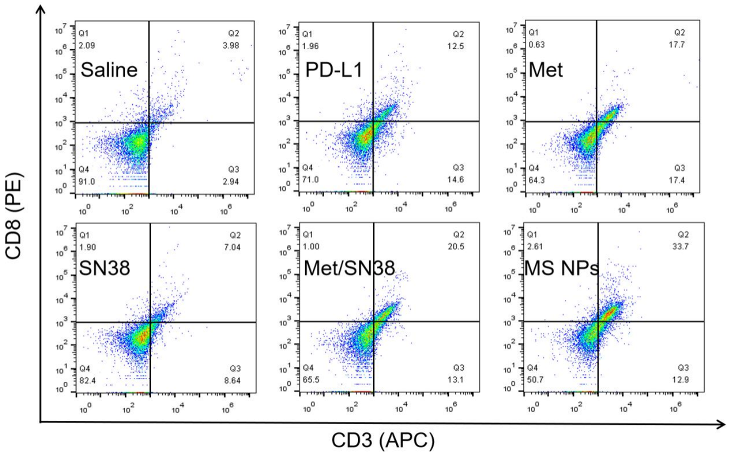

Figure S18. T cell immunofluorescent staining with flow cytometric analysis.

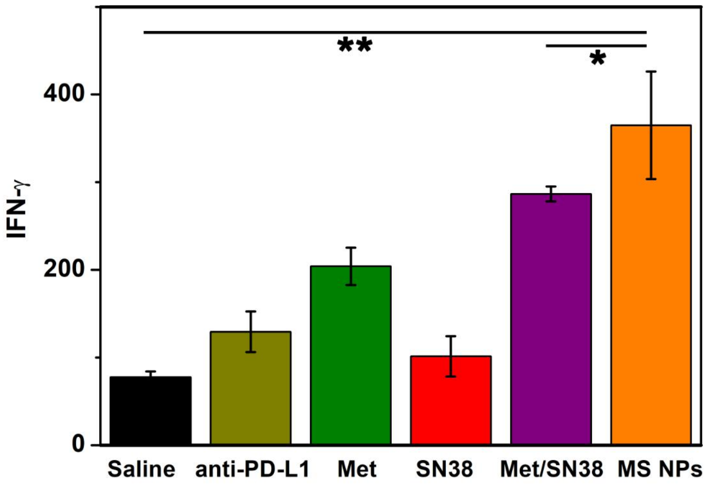

Figure S19. The levels of IFN-γ from saline, anti-PD-L1, Met, SN38, Met/SN38 and MS NPs treated mice.

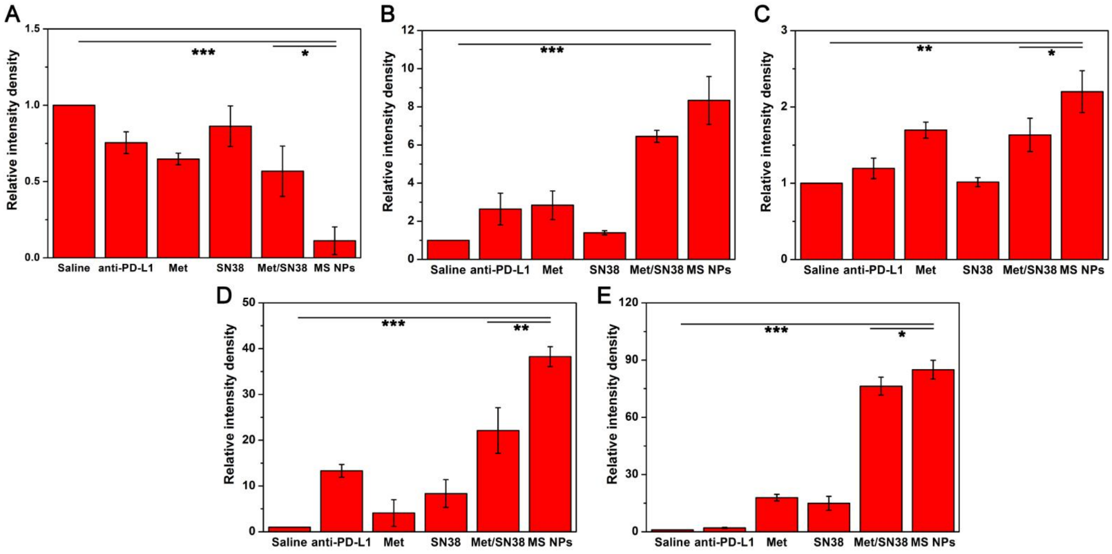

Figure S20. Quantification of positive staining of (A) PD-L1, (B) $CD8^+$ CTLs, (C) GzyB, (D) CCA3, and (E) TUNEL in Figure 5. The intensity density of each mark was calculated by Image J. The relative intensity density is equal to the intensity density of each sample divided by the that of the control group (saline-treated group).

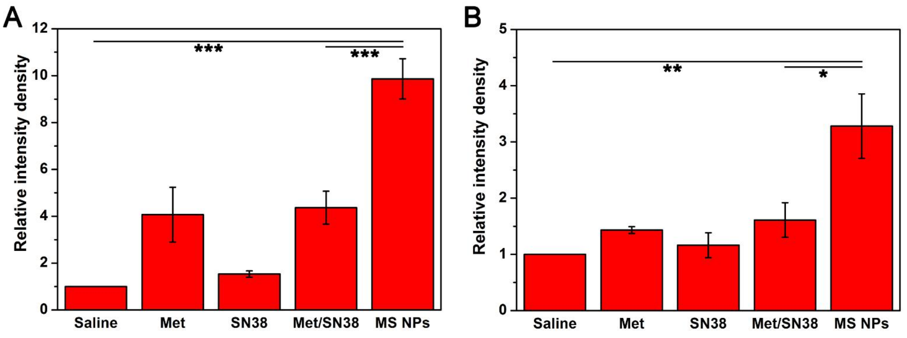

Figure S21. Quantification of positive staining of (A) $CD8^+$ CTLs and (B) GzyB in Figure 6d. The intensity density of each mark was calculated by Image J. The relative intensity density is equal to the intensity density of each sample divided by the that of the control group (saline-treated group).

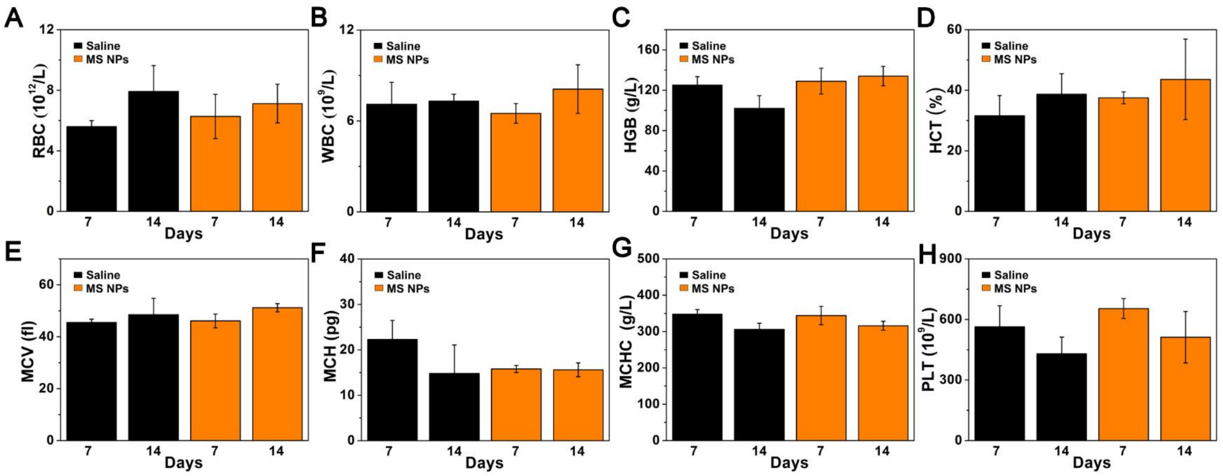

Figure S22. Haematology assay of saline and MS NPs treated mice at day 7 and 14.

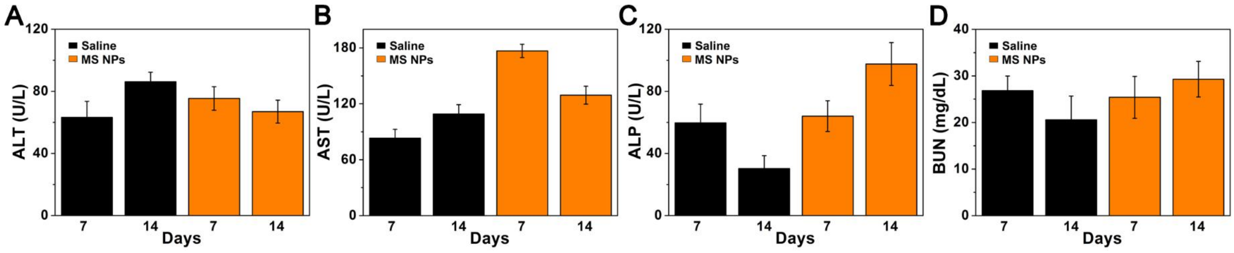

Figure S23. Blood chemistry assay of saline and MS NPs treated mice at day 7 and 14.
